# Incidence and Clinical Characteristics of Anaerobic Bacteremia at a University Hospital in Hungary: A 5-Year Retrospective Observational Study

**DOI:** 10.3390/antibiotics11101326

**Published:** 2022-09-28

**Authors:** Krisztina Kovács, Adrienn Nyul, Zsolt Lutz, Gyula Mestyán, Márió Gajdács, Edit Urbán, Ágnes Sonnevend

**Affiliations:** 1 Department of Medical Microbiology and Immunology, Clinical Centre, Medical School, University of Pécs, Szigeti út 12, 7624 Pécs, Hungary; 2 Department of Oral Biology and Experimental Dental Research, Faculty of Dentistry, University of Szeged, 6720 Szeged, Hungary

**Keywords:** anaerobes, bacteremia, bloodstream infections, MALDI-TOF, blood cultures, clinical study, *Bacteroides*, *Clostridium*

## Abstract

Strict anaerobes have been reported to account for 0.5–13% of episodes of bacteremia in the adult population, with a growing awareness among clinicians regarding anaerobic bacteremia, especially in patients with specific predisposing factors. The aim of our present study was to assess the incidence and clinical characteristics of anaerobic bacteremia during a 5-year period (2016–2020) at a tertiary care teaching hospital, and to compare our findings with other studies in Hungary. Overall, *n* = 160 strict anaerobes were detected, out of which, 44.4% (*n* = 71; 0.1% of positive blood cultures, 0.1/1000 hospitalizations, 3.3/100,000 patient days) were clinically significant, while *Cutibacterium* spp. accounted for 55.6% (*n* = 89) of isolates. Among relevant pathogens, the *Bacteroides/Parabacteroides* spp. group (32.4%; *n* = 23), *Clostridium* spp. (22.5%; *n* = 16) and Gram-positive anaerobic cocci (15.5%; *n* = 11) were the most common. The mean age of patients was 67.1 ± 14.1 years, with a male majority (59.2%; *n* = 42). A total of 38.0% of patients were affected by a malignancy or immunosuppression, while an abscess was identified in 15.5% of cases. A total of 74.7% (*n* = 53) of patients received antibiotics prior to blood culture sampling; in instances where antimicrobials were reported, anaerobic coverage of the drugs was appropriate in 52.1% (*n* = 37) of cases. The 30-day crude mortality rate was 39.4% (*n* = 28); age ≥ 75 years was a significant predictor of 30-day mortality (OR: 5.0; CI: 1.8–14.4; *p* = 0.003), while malignancy and immunosuppression, lack of anti-anaerobic coverage or female sex did not show a significant relationship with the mortality of these patients. Early recognition of the role played by anaerobes in sepsis and timely initiation of adequate, effective antimicrobial treatment have proven efficient in reducing the mortality of patients affected by anaerobic bacteremia.

## 1. Introduction

Anaerobic bacteria make up the vast majority of the microbiome of human skin, mucous membranes and gastrointestinal tract [1]. These microorganisms are characterized by a partial or complete intolerance to atmospheric oxygen, which may be explained by the lack of catalase, superoxide dismutase (SOD) and peroxidase enzymes, which are vital for the elimination of toxic reactive oxygen species [2,3]. With the exceptions of bite wound infections and gas gangrene, most anaerobic infections are from endogenous origins (from the site of normal colonization), leading to local abscess formation or invasive infections, which may be severe or life-threatening infections [4,5]. In the age of evidence-based medicine (EBM) and antimicrobial stewardship, strict sample collection and transport and proper anaerobiosis during culture are needed for the identification and antimicrobial susceptibility testing of anaerobes, to guide therapeutic decisions [6]. The susceptibility of clinically relevant anaerobes was thought to be predictable, allowing for safe empiric therapy with the relevant anti-anaerobic drugs; resistance rates in most anaerobic bacteria showed a considerable increase in the last 20–30 years [7]. Thus, rapid and accurate species-level identification of anaerobes aids clinicians to administer the best care for their patients, leading to significantly reduced morbidity and mortality rates and hospital stays [8]. However, even today, anaerobic bacteria are some of the most neglected, unrecognized pathogens, as their cultivation requires extensive microbiological experience, and many facilities (especially in developing countries) may not have the equipment (e.g., anaerobic chambers) that allow for proper anaerobiosis [9]. As a result, failure to isolate or accurately identify these bacteria may lead to inappropriate, unnecessary use of broad-spectrum antibiotics, subsequently leading to a worsening antimicrobial resistance situation [10].

There is considerable variation in the reported prevalence of anaerobic bacteria in bacteremia, which may be explained by different standard operating procedures in clinical practice, the healthcare institution in question (e.g., primary vs. tertiary), patient population and the capabilities of the diagnostic laboratory [11]. In addition, the need for performing blood cultures for anaerobic bacteria is sometimes contested, especially by clinicians, due to their opinion that the presence of anaerobes in blood may easily be suspected/predicted, based on the patients’ characteristics, and if the source of infection is known to be an anatomical area with a rich anaerobic flora [12]. Anaerobes have been reported to account for 0.5–13% of episodes of bacteremia in the adult population (before the introduction of various prophylactic measures, their prevalence was as high as 20%) [13]. It is crucial to establish early and effective therapy for patients affected by anaerobic bacteremia, as this condition has a considerable mortality rate (15–55%) [11,13]. There is, however, a growing awareness of the role of anaerobes in bacteremia, especially in those patients who have specific predisposing factors (e.g., advanced age, immunosuppression, cancer, previous surgical intervention) [14]. There is a scarce amount of research comparing the epidemiological features of anaerobic bacteremia in a multicenter fashion or within a country, and many of these comparisons are limited by the use of different operating standards and the laboratory methodologies used. The primary aim of our present study was to assess the incidence and clinical characteristics of anaerobic bacteremia during a 5-year period in a tertiary care university teaching hospital in the Southern Transdanubia region of Hungary. In addition, our secondary aim was to compare our findings to our previous study in the Southern Great Plain region of Hungary [15]; the comparison of data in these two studies is facilitated by a similar study setting and duration, similar patient population and the same instrumentation used for microbiological identification.

## 2. Results and Discussion

During the 5-year study period, on average, 11,548.6 ± 253.6 blood culture bottles were processed at the the Department of Medical Microbiology and Immunology per year (*n* = 57,743 overall), out of which, 18.6% (*n* = 10,759) were culture positive (including both contaminants and clinically relevant isolates). Overall, *n* = 160 strict anaerobes were detected, out of which, *Cutibacterium* spp. accounted for 55.6% (*n* = 89) of isolates. Based on our criteria, all of these isolates were considered as contaminants; therefore, they were excluded from the analysis of clinical data. Clinically relevant anaerobes constituted 0.1% (*n* = 71) of all processed blood cultures (0.7% of all positive blood cultures), representing 1.2 isolates/1000 blood culture bottles, 0.1 isolates/1000 hospitalizations and 3.3 isolates/100,000 patient days, respectively. Details regarding the primary epidemiological characteristics of anaerobic bacteremia in the present and comparator study are presented in Table 1. The mean TAT (turnaround time) for the processing, identification and reporting of anaerobic pathogens in the 5-year period was 7.8 ± 3.2 days.

The demographic characteristics of patients affected by anaerobic bacteremia in the present and comparator study are shown in Table 2; a male dominance (59.2%; *n* = 42) was observed, while the mean age of patients was 67.1 ± 14.1 years. A total of 29.6% (*n* = 21) of patients were affected by a solid tumor or a hematological malignancy (the most common being colon adenocarcinoma, in *n* = 6 cases), in addition to 8.5% (*n* = 6) of patients who were immunosuppressed due to other illnesses or organ transplantation. Sepsis, pneumonia, cardiovascular illness or intervention, and pancreatitis were described in 11.3% (*n* = 8), 11.3% (*n* = 8), 11.3% (*n* = 8) and 4.2% (*n* = 3) of patients, respectively. An abscess was identified in 15.5% of cases (*n* = 11, out of which, *n* = 10 were located in the abdomen or the rectum), 5.6% (*n* = 4) had severe decubitus ulcers or gangrene, while in 4.2% (*n* = 3) of patients, perforations in the large intestines were noted. Polymicrobial bloodstream infections were observed in 12.7% (*n* = 9) of affected patients; *Escherichia coli* was the co-pathogen in *n* = 3 cases, while *Proteus mirabilis* was the co-pathogen in *n* = 1 case.

A total of 74.7% (*n* = 53) of patients received antibiotics prior to blood culture sampling; in instances where antimicrobials were reported, anaerobic coverage of the drugs was appropriate in 52.1% (*n* = 37) of cases (i.e., the patient received antibiotics that are effective against anaerobes). The 30-day crude mortality rate was 39.4% (*n* = 28) among patients with clinically significant anaerobic bacteremia. Age ≥ 75 years was a significant predictor of 30-day mortality (OR: 5.0; CI: 1.8–14.4; *p* = 0.003), while malignancy (OR: 0.5; CI: 0.2–1.5; *p* = 0.229), malignancy and immunosuppression (OR: 0.9; CI: 0.3–2.3; *p* = 0.746), lack of anti-anaerobic coverage (OR: 0.5; CI: 0.2–1.2; *p* = 0.200) and female sex (OR: 1.9; CI: 0.7–4.9; *p* = 0.208) did not show a significant relationship with the mortality of patients.

The detailed species distribution of anaerobic isolates in clinically relevant anaerobic bacteremia is presented in Table 3, while a comparative representation of data with the reference study and other literature findings is shown in Table 4. Overall, the majority of anaerobic pathogens were from the *Bacteroides/Parabacteroides* spp. group (32.4%; *n* = 23) and *Clostridium* spp. (22.5%; *n* = 16); the distribution among Gram-positive and Gram-negative anaerobes was almost equal (36 vs. 35).

Anaerobic bacteria are important constituents of the normal human microbiota, in addition to having the potential of becoming relevant pathogens when displaced into normally sterile anatomical sites in the body [16]. These bacteria may be important etiological agents in bloodstream infections and/or other severe invasive infections, although their reported prevalence is relatively low (which may also be explained by a bias of under-reporting) [17]. In the present study, *n* = 71 cases of clinically relevant anaerobic bacteremia were identified, over a period of 5 years (2016–2020); this corresponded to 0.7% of all positive blood cultures or 1.2 isolates/1000 blood culture bottles, which is at the lower end of the range currently reported in the literature (0.5–13%) [11,13]. Similarly to other literature sources, *Cutibacterium* spp. accounted for the majority (>50%) of anaerobes in the study period, representing over half of the isolates both here and in our previous study [15]. In contrast to the comparator study from Szeged—which reported a 2.5-times higher prevalence of anaerobic bacteremia between 2013 and 2017, and a pronounced female dominance in affected patients—herein, male patients were more commonly affected. Our study population showed the characteristics of patients at risk for anaerobic bloodstream infections [18]; most of them were elderly, and over 38% of patients were affected by malignancy and/or immunosuppression (vs. 8% [15]). In line with our previous study (and in line with the summary of other literature reports), *Bacteroides*/*Parabacteroides* species (among Gram-negatives) and *Clostridium* spp. (among Gram-positives) were the most common clinically relevant pathogens detected (corresponding to 60–80% of the isolates, see Table 4), while a new species (*A. schaali*), not previously reported in the region, has also been described. GPAC, and Gram-positive and Gram-negative rods other than clostridia and *Bacteroides/Parabacteroides* spp., represented the minority of anaerobic isolates, which, barring a report from Sweden [19], is in line with the literature. Similarly to previous findings from the region, Gram-negative anaerobic cocci were uncommonly isolated, with a 2–3% share in anaerobic bloodstream infections [20]. Although in the study from Szeged, Gram-positive anaerobic bacteria dominated during the 5-year period (61.1% vs. 49.3% in the present study), we were faced with the dominance of Gram-negative anaerobes (50.7% vs. 38.9% in Szeged). One of the main differences between the two clinical centers was the isolation rate of *Fusobacterium* spp.; in the present study, their rate was 9.9% (*n* = 7 patients), while this was only 1.2% (*n* = 2 patients) in the previous study [20]. Out of these patients, four had a malignant underlying disease (lymphoma, colon carcinoma, supraglottic squamous cell carcinoma and carcinoma of the appendix), with three out of seven patients recovering.

Many studies have called into question the relevance of blood cultures for anaerobic bacteria; on one hand, several papers reported a considerable and sustained decrease in the prevalence of anaerobic bacteremia [21,22]. In addition, clinical studies have shown that patients’ characteristics (e.g., the presence of neutropenia) were indicative of anaerobic bacteremia, and clinical cure was achieved without the need of blood cultures, as the source of infection (most commonly from the gastrointestinal region, the urogenital region or the oropharynx) was obvious [23,24]. For example, De Keukeleire et al. followed the epidemiology of anaerobic bacteremia for a 10-year period [25] and found a decreasing trend from 2004 to 2008 and 2009–2013, with 17.3/100,000 patient days to 13.7/100,000 patient days, and 1.3/1000 patients to 0.9/1000 patients, respectively. Similarly, Morris et al. showed that in their study, the source of bacteremia was evident in >80% of cases [26]. Anaerobic bacteremia is almost invariably secondary to a focal primary infection; therefore, the anaerobic strains recovered often depend on the portal of entry and the underlying disease of the patients [27]. Nevertheless, both of the abovementioned points were contested by other reports, highlighting that: (i) with the increasing prevalence of patients with cancer/immunosuppression, and with the increasing use of invasive medical technologies, a parallel increase in anaerobic bacteremia was observed [28], (ii) misinterpretation of the infection source or clinical findings may affect outcomes [29], (iii) novel, previously unreported pathogens are increasingly being described as clinically significant [30], and (iv) without isolation and susceptibility testing, therapy may fail if an antibiotic-resistant isolate is the cause of the infection [31].

The 30-day crude mortality rate for our patient sample was 39.44%; advanced age was a risk factor for mortality, while no significant associations were shown with the other studied correlates. These findings were similar to the study of Kim et al., where cardiovascular disease was a main risk factor for mortality [32], and Blairon et al., where overall mortality was 13%, and the fatal outcome was mainly influenced by severe underlying diseases, but not by antimicrobial coverage or the causative pathogen [33]. On the other hand, the study of Salonen et al. highlighted the importance of appropriate anti-anaerobic coverage in anaerobic bacteremia; in their study, only 50% of patients received correct antibiotics initially, while the mortality rates among patients with appropriate therapy (or therapy that had been changed after culture results) vs. the ones without coverage for anaerobes were 18%/17% vs. 55%, respectively [29]. In the study of Ramos et al., mortality attributable to anaerobic bacteremia was 32.0%, where septic shock, kidney failure, failure to perform drainage and inappropriate antimicrobial therapy were the principal risk factors for mortality [34]. Finally, in a recent publication by Cobo et al., *Bacteroides* (43.9%), *Clostridium* (24.1%) species and GPAC (15.6%) were among the most common pathogens in anaerobic bacteremia, with an attributable mortality rate of 20%. In addition, their study highlighted the considerable increase in nonsusceptibility rates of anaerobes against commonly used “anti-anaerobic” antimicrobials [35]. Some key limitations need to be addressed: (i) the retrospective, single-center study design, leading to a relatively low number of clinically relevant infections to be analyzed; (ii) selection bias, as the study population originated from a tertiary care center, corresponding to patients with more severe conditions or underlying illnesses; (iii) the lack of phenotypic antibiotic susceptibility data or genetic characterization for the respective isolates; and (iv) the lack of the patients’ laboratory results (e.g., white blood cell count, coagulation parameters, liver enzymes, inflammatory markers or other biomarkers) included in the analysis.

## 3. Conclusions

Despite their relatively low prevalence, anaerobic bacteria may be important causative agents of severe infections in patients with characteristic underlying conditions, which are often associated with a high mortality rate. Based on these known facts, it would be extremely important to review, analyze and compare the trends in the occurrence of anaerobic bacteremia locally. Overall, early recognition of the role played by anaerobes in sepsis and timely initiation of adequate, effective antimicrobial treatment, recognition of the source of infection and proper control have proven efficient in reducing the mortality of patients affected by anaerobic bacteremia. The increasing awareness of clinicians regarding anaerobes may be partly due to the emergence of effective microbial identification methods—such as matrix-assisted laser desorption ionization time-of-flight mass spectrometry (MALDI-TOF MS)—which have allowed the identification of these pathogens in a clinically relevant time frame. MALDI-TOF MS should replace all other identification techniques for the routine identification of anaerobes in clinical microbiology laboratories. Developments such as MALDI-TOF MS—in addition to the introduction of 16S rRNA sequencing platforms, as a relevant identification method in routine laboratories—will ensure timely and accurate diagnostics for patients affected by anaerobes, and may also allow for the rapid detection of resistance mechanisms. The cooperation of microbiology laboratories with the clinical suspicion of the treating physicians will safeguard the appropriate selection of antimicrobials for these infections.

## 4. Materials and Methods

### 4.1. Study Design, Collection of Data

The present retrospective observational study was carried out on the basis of microbiological and clinical patient data, collected corresponding to the 5-year time period between 1 January 2016 and 31 December 2020. The clinical records of all patients with positive BCs in the study period were reviewed retrospectively. During the study period, the Department of Medical Microbiology and Immunology provided routine diagnostic microbiological services at the University of Pécs (PTE) Clinical Centre (CC). The CC is a 1725-bed tertiary care university teaching hospital in the Southern Transdanubia region of Hungary, serving as a primary facility to more than 800,000 inhabitants (~17% of the population is aged 65 years or older), according to the National Health Insurance Fund data for 2020 [36,37]. The CC maintains four adult intensive care units (ICUs; cardiology, hematology, trauma and surgical) and two pediatric ICUs (pediatric and neonatal), with different profiles.

Microbiological culture results for anaerobic bacteremia for adult patients (≥18 years) were collected via an electronic search of the laboratory information system (LIS) of the Department of Medical Microbiology and Immunology, related to the 5-year study period. Data were collected for samples originating from inpatient departments and the emergency department, while outpatient clinics were excluded. According to the case definition used in the literature, isolates were considered separate if they occurred more than 14 days apart, otherwise they were excluded [19]. Data were collected regarding the age, sex, anamnestic data (e.g., underlying conditions, presence of known immunosuppression or cancer, presence of a known abscess or focal infection), previous antimicrobial therapy and the 30-day crude mortality rate of the affected patients; data on turnaround times (TAT) were also noted in each case. Although *Cutibacterium* spp. isolates are not usually considered as causative agents of bacteremia, their relevance was ascertained based on clinical patient data, according to the criteria described previously [15]. If *Cutibacterium* spp. was not considered clinically relevant, clinical data corresponding to those cases were excluded.

### 4.2. Sample Processing, Microbiological Identification

Blood culture sampling was performed at the request of the attending physicians, who were responsible for the principal decisions concerning the diagnosis and treatment of patients. Blood culture processing was carried out according to national and international recommendations [38,39]. Clinicians routinely used parallel aerobic and anaerobic blood culture bottles in pairs, with most blood cultures ordered as two sets, with one aerobic and one anaerobic bottle per set. Blood cultures were analyzed in the laboratory by the BD Bactec^TM^ automated system (Becton Dickinson, Franklin Lakes, NJ, USA), following inoculation of 5–10 mL of blood into aerobic and anaerobic bottles, respectively. Incubation of the blood culture bottles was performed for 7 days (21 days, if endocarditis was suspected) with constant shaking, according to the manufacturer’s instructions.

Samples from positive bottles were plated onto Schaedler agar (bioMérieux, Marcy l’Etoile, France) containing 5% *v*/*v* horse blood, haemin and Vitamin K_1_ to isolate anaerobic bacteria; plates were incubated in an anaerobic environment in an atmosphere of 90% N_2_, 5% H_2_ and 5% CO_2_, with the aid on an anaerobic chamber (Concept 400 anaerobic incubator, Biotrace International Plc., UK). The incubation time generally lasted for 2–5 days at 37 °C [40]. Identification of the isolates was carried out with matrix-assisted laser desorption ionization time-of-flight mass spectrometry (MALDI-TOF MS), using a microFlex LT Biotyper (Bruker Daltonics, Bremen, Germany), the MALDI Biotyper RTC 3.1 software (Bruker Daltonics, Germany) and the MALDI Biotyper Library 3.1 for spectra analysis. To facilitate successful identification, a formic acid extraction step was included before the measurements. The sample preparation methodology and the technical details of MS measurements were described elsewhere [41]. Genus-level identification was considered reliable for *log*(score) ≥ 1.7, while the value for species-level identification was *log*(score) ≥ 2.0.

### 4.3. Statistical and Comparative Analysis

Descriptive statistical analysis (including means with ranges and percentages to characterize the data) was performed using Microsoft Excel 2013 (Microsoft Corp. Redmond, WA, USA). The relationships between the 30-day crude mortality rate and other relevant study correlates were expressed as odds ratios (OR ± 95% confidence interval [CI]), which were calculated by SPSS software version 22 (IBM Corp., Endicott, NY, USA). *p* values < 0.05 were considered statistically significant.

For the sake of comparison, and to present the results in a national context, the findings of this study were directly compared to a similar retrospective epidemiological study regarding anaerobic bacteremia, performed by the authors in the Southern Great Plain of Hungary (at the Albert Szent-Györgyi Clinical Center, University of Szeged) [15]. The bases of the comparison are: the same study design and duration (2013–2017), similar demographic characteristics of the population, similar tertiary care university hospital setting, the same guidelines used for sampling and the same instrumentation used for microbiological identification.

### 4.4. Ethical Considerations

The study was conducted in accordance with the Declaration of Helsinki, and national and institutional ethical standards. Ethical approval for the study protocol was obtained from the Human Institutional and Regional Committee for Research Ethics, University of Pécs (registration number: KK-164-2/2021).

## Figures and Tables

**Table 1:** Comparison of study settings and primary results of the present study with our previous comparator study.

	Present Study	Gajdács et al. [15]
**Study location**	University of Pécs (PTE) Clinical Centre (Pécs, Hungary)	Albert Szent-Györgyi Clinical Center, University of Szeged (Szeged, Hungary)
**Study period**	2016–2020	2013–2017
**Hospital bed count**	Acute: 1705 Chronic: 20	Acute: 1465 Chronic: 355
**Population served**	~800,000 patients	~600,000 patients
**Number of hospitalized patients/year** **(Average ± SD)**	100,461 ± 9623	84,438 ± 1866
**Number of blood culture bottles processed during the study period**	57,743	116,371
**Percentage of positive blood culture bottles for aerobic and anaerobic bacteria overall** **(including contaminants)**	18.6 ± 2.1%	10.5 ± 0.3%
**Percentage of positive blood culture bottles for clinically relevant anaerobes** **(Percentage of positive blood culture bottles for all anaerobes)**	0.1 ± 0.03% (0.3 ± 0.03%)	0.2 ± 0.02% (0.4 ± 0.1%)
**Number of clinically relevant anaerobic isolates in bacteremia** **(Number of all anaerobic isolates in bacteremia)**	71 (160)	176 (423)
**Number of clinically relevant anaerobic isolates/1000 hospitalizations** **(Number of all anaerobic isolates/1000 hospitalizations)**	0.1 (0.3)	0.4 (1.0)
**Number of clinically relevant anaerobic isolates/100,000 patient days** **(Number of all anaerobic isolates/100,000 patient days)**	3.3 (7.4)	8.5 (20.6)
**Methods used for microbial identification**	MALDI-TOF MS (Bruker Daltonics), extraction with formic acid before measurements	
**Blood culture detection system**	BD Bactec^TM^ (Becton Dickinson)	BacT/Alert 3D (bioMérieux)

**Table 2:** Demographic characteristics of patients affected by anaerobic bacteremia in the present study and in our previous comparator study.

Study Year	Present Study	Gajdács et al. [15]
**Number of affected patients**	71	187
**Male-to-female ratio**	1.45	0.60
**Mean age [year ± SD]**	67.1 ± 14.1	71.9 ± 16.7
**Age range [years]**	25–97	18–102

**Table 3:** Detailed species distribution of clinically relevant anaerobic isolates from anaerobic bacteremia in our study (2016–2020).

Study Year	2016	2017	2018	2019	2020	Overall
**Number of Affected Patients**	**15**	**10**	**19**	**12**	**15**	**71 (100%)**
**Gram-positive, spore-forming anaerobic rods**	**3**	**3**	**6**	**0**	**4**	**16 (22.5%)**
*Clostridium* sp. (genus level)	1					1
*C. baratii*					1	1
*C. hathewayi* (*Hungatella hathewayi*)			1			1
*C. paraputrificum*					1	1
*C. perfringens*		1	4		1	6
*C. ramosum*		1	1		1	3
*Paeniclostridium sordellii*	2					2
*C. septicum*		1				1
**Gram-positive, non-spore-forming anaerobic rods**	**0**	**1**	**1**	**5**	**1**	**8 (11.3%)**
*Actinotignum schaali*				2		2
*Actinomyces naeslundii*			1			1
*A. neuii*		1				1
*A. odontolyticus (Schaalia odontolytica)*				2	1	3
*A. turicensis (Schaalia turicensis)*				1		1
*Eggerthella lenta*					2	2
*Lactobacillus fermentum*					1	1
*Weissella viridescens*					1	1
**Gram-positive anaerobic cocci (GPAC)**	**5**	**1**	**3**	**0**	**2**	**11 (15.5%)**
*Anaerococcus octavius*			1			1
*A. tetradius*			1			1
*Parvimonas micra*		1			2	3
*Peptinophilus harei*			1			1
*Peptococcus niger*	2					2
*Peptostreptococcus* sp. (genus level)	3					3
**Gram-negative anaerobic rods**	**7**	**5**	**8**	**6**	**8**	**34 (47.9%)**
*Bacteroides* sp. (genus level)	5	1				6
*B. caccae*				1		1
*B. fragilis*	1	3	2	2	4	12
*B. faecis*	1					1
*B. thetaiotaomicron*			1		1	2
*Fusobacterium* sp. (genus level)				1		1
*F. nucleatum*			2		3	5
*F. periodonticum*			1			1
*Parabacteroides distasonis*				1		1
*Prevotella* sp. (genus level)			1			1
*P. bivia*		1				1
*P. intermedia*				1		1
*P. melaninogenica*			1			1
**Gram-negative anaerobic cocci**	**0**	**0**	**1**	**1**	**0**	**2 (2.8%)**
*Veillonella parvula*			1	1		2

**Table 4:** Percentage distribution of anaerobes in the two respective studies and in comparison with the literature findings.

Anaerobic Isolates	Percentage of Anaerobes According to Literature Data [11,13]	Present Study	Gajdács et al. [15]
***Cutibacterium*** spp. *	**30–80%** **(of anaerobes isolated)**	**55.6% (*n* = 89)**	**54.0% (*n* = 247)**
All other isolates excluding *Cutibacterium* spp.:			
**Gram-negative anaerobes**		**50.7% (*n* = 36)**	**38.9% (*n* = 69)**
*Bacteroides/Parabacteroides* spp.	**26–75%**	32.4% (*n* = 23)	34.2% (*n* = 54)
*Fusobacterium* spp.	**4–15%**	9.9% (*n* = 7)	1.2% (*n* = 2)
*Prevotella* and *Porphyromonas* spp.	**0.5–10%**	5.6% (*n* = 4)	1.2% (*n* = 2)
*Veillonella* spp.	**0.5–2%**	2.8% (*n* = 2)	2.3% (*n* = 4)
**Gram-positive anaerobes**		**49.3% (*n* = 35)**	**61.1% (*n* = 107)**
*Clostridium* spp.	**8–46%**	22.5% (*n* = 16)	33.3% (*n* = 59)
Gram-positive anaerobic cocci (GPAC)	**8–20%**	15.5% (*n* = 11)	12.0% (*n* = 21)
Gram-positive non-spore-forming rods (excluding: *Cutibacterium* spp.)	**0.5–14%**	11.3% (*n* = 8)	15.8% (*n* = 27)

* Usually not considered clinically relevant (contaminants).

## Data Availability

All data generated during the study are presented in this paper.
